# Metal-matrix nanocomposites under compressive loading: Towards an understanding of how twinning formation can enhance their plastic deformation

**DOI:** 10.1038/s41598-020-66696-1

**Published:** 2020-06-16

**Authors:** A. Kardani, A. Montazeri

**Affiliations:** Computational Nanomaterials Lab (CNL), Faculty of Materials Science and Engineering, K.N. Toosi University of Technology, Tehran, Iran

**Keywords:** Nanoscale materials, Structural materials, Theory and computation, Atomic and molecular physics, Computational science

## Abstract

Recently, Cu-Ag nanocomposites (NCs) have been extensively used as medical implants and surgical instruments due to their antibacterial properties. Consequently, mechanical behavior analysis of these NCs is of crucial importance with emphasis on their plastic deformation mechanisms. From the materials science perspective, dislocations slip at the room temperature and high strain rates conditions is hindered. However, copper and silver, as two metals with low stacking fault energy are prone to twin formation. Since microstructural changes in these nanostructured composites occur at the atomic scale, molecular dynamics (MD) simulation is undoubtedly a great tool to use. Accordingly, in the present research, first, the deformation mechanism of perfect copper-silver NCs under uniaxial compression is deeply analyzed employing MD. This is followed by inspection of the voids effect on their plastic deformation process. The results show that twinning is the dominant mechanism governing their deformation under uniaxial compressive loading conditions. It is revealed that twins are created by the conversion of internal stacking faults to their external counterparts. Also, investigation of the microstructural evolution demonstrates that the presence of voids within NC samples provides new sites for nucleation of Shockley dislocations in addition to the interface zone. Finally, to address the effect of interfacial coherency on the results, copper-based NCs infused with gold and nickel nanoparticles are also thoroughly examined.

## Introduction

In recent years, metal matrix nanocomposites (MMNCs) have been widely used in health, transportation, electronics, and aerospace industries^1^, which is attributed to their outstanding physical and mechanical characteristics compared to the base metal matrix. Among these, copper-silver NCs can significantly improve the performance of biomedical implants due to their antibacterial effects^2,3^. It is worth mentioning that the use of nanocomposites for this goal may face several challenges. For example, an implant as a replacement of the damaged tissue should possess mechanical properties similar to those of the bone structure. Otherwise, under external loading conditions, deformation occurring on the implant is really different from that of the bone leading to the so-called implant loosening^4–6^. Accordingly, the analysis of their mechanical behavior and deformation mechanism is a focal point of research. From the materials science perspective, at the atomic scale, plastic deformation of nanocrystalline materials is achieved through twinning and/or slip of dislocations^7^. Although both of these mechanisms change the mechanical properties of the sample, however, their influence on the microstructural evolution can be completely different. To be more specific, dislocations slip creates local deformations, whereas twinning reorients the crystal structure with the cooperative movement of atoms. However, from the microscale point of view, since deformation by dislocations takes place in the whole material, plastic deformation occurs mainly through this mechanism^8^. One of the other factors limiting the twinning-based deformation is the fact that slip normally occurs in the discrete multiples of the atomic spacing. Meanwhile, the atom movements are much less than the mentioned parameter during the deformation twinning^9^.

Several factors affect the type of plastic deformation mechanism that occurred within the nanocrystalline metals. For example, in the case of metals with nano-sized grains, twinning is the governing mechanism, whereas with increasing the grain size, dislocation slip is more likely to happen^10^. It also depends on the type of crystal structure. In BCC and HCP structures, due to the lack of sufficient active slip systems, deformation occurs by the twinning process. Similar behavior has also been observed in the low stacking fault energy (SFE) metals having the FCC structure such as copper, silver, and aluminum, which is ascribed to the inverse relationship between the SFE and twinning behavior^11–13^. Additionally, it has been demonstrated that twinning-based deformation is favored at higher strain rates and lower temperature conditions^14,15^.

Concerning the plastic deformation behavior of copper/silver nanocomposites, a key question naturally arises: While a good consistency is predicted for slip planes and slip directions of these two metals owing to their similar structure, why their composites exhibit a different behavior? Finding the possible answer to this question is the main aim of the present work. In this context, most previous studies have been devoted to analyze the plastic deformation behavior of MMNCs in the presence of the second phase. Among these, it has been demonstrated that the formation of stacking fault regions within the bulk matrix has a direct correlation with the geometrical features of the additive phase and its distribution^16^. Carrying out molecular dynamics (MD) simulations, Weng *et al*.^17^ have also shown that the interfacial area is one of the most important sites for the nucleation of dislocations and therefore, can noticeably affect the mechanical behavior of nanocomposites. Regarding this issue, Pogorelko *et al*.^18^ demonstrated that copper nanoparticles form a semi-coherent interface with the aluminum matrix and as a result, efficient load transfer cannot be achieved from the matrix to the embedded copper nanofillers. However, the interfacial region can play a different role in the plastic deformation behavior of MMNCs. For example, in the study of Buehler *et al*.^19^, it has been observed that the niobium/copper interface would stop the slip of dislocations leading to the enhanced yield strength and morphological stability of the nanocomposite samples at the higher temperature conditions.

An important issue regarding the biomedical applications of these NCs is the adhesion of body cells to the implants, which improves the strength of the implant/tissue interface. It is worth mentioning that this phenomenon could be drastically enhanced through the formation of nanovoids within the bulk matrix^20^. However, the formation of these porosities is considered to have negative effects on the mechanical properties. This is attributed to the creation of partial dislocations in the interfacial region as depicted by Zhao *et al*.^21^ during the analysis of the defected single crystalline copper sample under tensile loading conditions. This arises because voids can resemble the dislocation generation sources due to the locally induced stress concentrations in their vicinity^21^. This issue was previously addressed by Zhan *et al*.^22^ for copper nanowires analyzed under uniaxial tensile test. It was concluded that porosities can act as new sites for the creation of dislocations, which in turn deteriorates the mechanical characteristics of these nanostructures.

In recent years, extensive research has been conducted to examine the role of interfacial area, type and location of voids, and geometrical features of the second phase on the mechanical behavior of MMNCs under tension loading conditions. However, the influence of the above-mentioned phenomena on the mechanical response of these nanocomposites to the other loading conditions has rarely been considered using atomic-scale simulation techniques. In particular, to our knowledge, no study has been devoted to examine the mechanical characteristics of defected Cu/Ag nanocomposites under compression. Therefore, in this study, we utilize MD simulation to see the effects of voids content on the compression behavior of copper-based nanocomposites embedded with Ag NPs. Additionally, the underlying plastic deformation mechanisms are introduced and compared to those activated in the tensile loadings. It is worth mentioning that since spherical Ag nanoparticles have more antimicrobial effects compared to the other geometrical features such as nanoplatelets, nanorods, and etc^23–25^., in the present work, this special shape of Ag NPs has been considered in our models. This paper is organized as follows. The simulation details and sampling techniques are denoted in Section 2 along with introducing the crystal structure analysis tools including Common Neighbor Analysis (CNA), Centrosymmetry Parameter (CSP), and Dislocation Extraction Algorithm (DXA). The most important findings of this work are presented in Section 3 with emphasis on the mechanisms governing the plastic deformation of MMNCs under compression. Section 4 contains the concluding remarks.

## Molecular dynamics model

As mentioned before, this paper is aimed to assess the nanovoids effect on the mechanisms governing the plastic deformation of Cu/Ag nanocomposites subjected to uniaxial compressive loadings. In the following, MD parameters, samples preparation method, and the most common techniques implemented to analyze the crystal structure evolutions within the samples are introduced.

### Parameters of MD simulation

**Figure 1:**
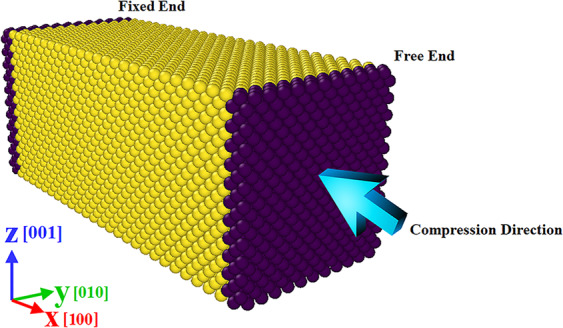
Schematic diagram of the uniaxial compressive test setup. Taken from OVITO (version 2.9 27Jul2017 https://www.ovito.org).

Simulations were carried out using LAMMPS (version 23Oct2017 https://lammps.sandia.gov)^26^. In line with the previous studies of various copper-silver systems^27–29^, the EAM potential was employed as the interaction model. This many-body potential has been successfully utilized to analyze the dislocations and stacking fault regions in metals, alloys, and MMNCs^1^. It should be pointed out that in the present work, the EAM potential developed by Williams *et al*.^30^ was utilized to mimic the interactions within the Cu-Ag samples. This version of EAM has been successfully employed to reproduce the lattice parameter, cohesive energy, elastic constants, lattice-defect energies, phonon frequencies, thermal expansion, and stacking faults energy of the Cu–Ag compounds. Additionally, comparing the simulated data with the experimental measurements, it has been shown that the Cu-Ag phase diagram can also be correctly predicted utilizing this potential, which demonstrates the validity of the EAM potential used in the current study. For each simulation, at first, it is required to relax the structure so that the energy of the whole system stabilizes at the lowest possible value. For this purpose, setting the temperature at 300 K, analysis of 200 ps at NVT ensemble was initially performed for all samples. Based on our previous study^31^, the value of 2 fs was considered as the time-step, which has proven to provide the accurate data at a reasonable computational cost for these systems. For controlling the temperature at the desired value, the Nose-Hoover thermostat was employed. After assigning the initial atomic velocities by the Maxwell-Boltzmann distribution, the well-known velocity Verlet scheme was implemented to advance the system variables^32^. Following the relaxation, to apply the uniaxial compression load on each sample, the left part was kept fixed (See Fig. 1). Then, as described in^33,34^, the right side was incrementally compressed to achieve the targeted strain level. For this purpose, in each step, the sample was undergone an axial displacement equal to 0.1 Å followed by the equilibration procedure for 600 ps. To eliminate the artificial surface effects on the results, periodic boundary conditions were considered in the lateral directions. Also, a shrink-wrapped boundary condition was assigned on the loading direction to let the atoms move freely, resulting in the imposed incremental displacement. To visualize the results, OVITO software (version 2.9 27Jul2017 https://www.ovito.org) was utilized^35^.

### Sample construction

**Figure 2:**
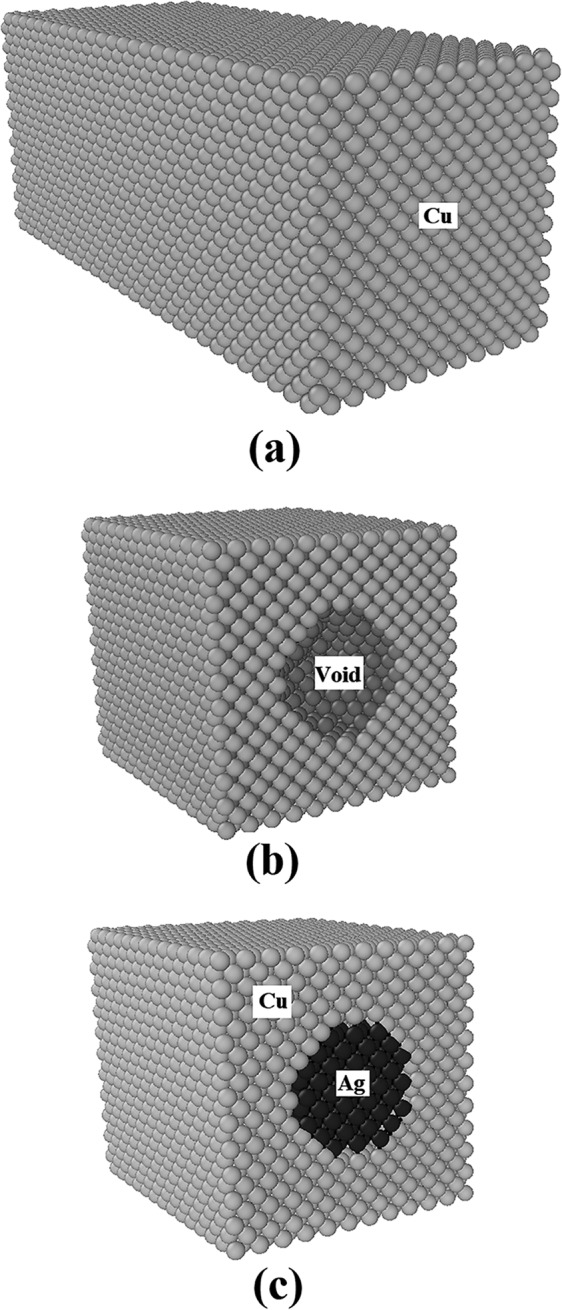
Three steps in the NC model construction including the design of a porous rectangular-shaped copper matrix and replacement of an additive silver phase. Taken from OVITO (version 2.9 27Jul2017 https://www.ovito.org).

**Figure 3:**
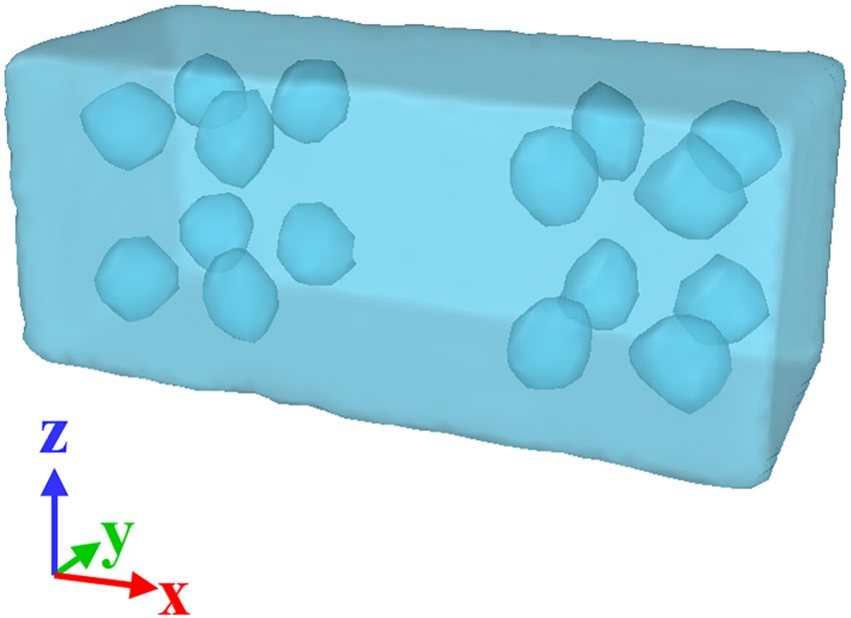
Illustration of voids positioning in the sample with 5.0% porosity. Taken from OVITO (version 2.9 27Jul2017 https://www.ovito.org).

Based on the Pogorelko and Mayer method^18^, nanocomposite samples were built in two steps as schematically shown in Fig. 2. Initially, a 10 × 4 × 4 nm single crystal copper with a central hole of 2 nm in diameter was constructed as the bulk matrix. Subsequently, the silver nanoparticle with a volume fraction (VF) of 2.6% was introduced to resemble the nanocomposite sample. To explore the voids effect on the results, different porous NC models were built. Characteristics of these samples have been summarized in Table 1. Among them, the case having 5.0% voids is depicted in Fig. 3.

**Table 1:** Input data for MD analysis of the NC samples having different voids content.

Voids content (%)	Number of voids	Diameter of voids (nm)
1.3	4	1
2.5	8	1
5.0	16	1

### Crystal structure analysis tools

To distinguish the various crystalline structures within the samples, CNA method was utilized. In this approach, the local neighbors of each atom are inspected based on the interatomic distances. The results are encoded as integer values. For example, FCC and HCP structural types are identified with numbers 1 and 2, respectively. Also, 3 and 4 are the indicators of BCC and icosahedral structures, and 5 is utilized for the free surfaces, sample edges and other unknown structures. In this technique, a threshold distance criterion is used to determine whether a pair of atoms is bonded or not. The input cut-off radius for any crystal lattice can be chosen using equations (1) to (3) ^36,37^:

1 $${r}^{FCC}=\frac{1}{2}\left(\frac{\sqrt{2}}{2}+1\right){a}_{0}=0.854{a}_{0}$$

2 $${r}^{BCC}=\frac{1}{2}(\sqrt{2}+1){a}_{0}=1.207{a}_{0}$$

3 $${r}^{HCP}=\frac{1}{2}\left(1+\sqrt{\frac{4+2{(1.63)}^{2}}{3}}\right){a}_{0}=1.38{a}_{0}$$

In these equations, ${{\rm{a}}}_{0}$ stands for the lattice parameter of the unit cell.

Additionally, the CSP method (Eq. 4) was employed for the identification of dislocations, stacking faults and twinning boundaries. This parameter provides a measure for deviation of a typical atom $i$ from the symmetric crystal structure. For an FCC crystal, CSP is given by^38^:

4 $$CS{P}_{i}^{FCC}=\mathop{\sum }\limits_{\beta =1}^{6}{|{\overrightarrow{R}}_{i,\beta }+{\overrightarrow{R}}_{i,\beta +6}|}^{2}$$

where ${\overrightarrow{R}}_{i,\beta }$ and ${\overrightarrow{R}}_{i,\beta +6}$ are vectors representing the 6 pairs of nearest neighbors in this type of crystal structure. Additionally, with respect to the crucial role of the dislocation density on the results and the underlying mechanisms, this parameter was computed through the DXA analysis^39^. Moreover, to identify the defects and crystal lattice type, classification of colors using the Pattern Matching Algorithm was utilized^40^.

## Results and discussion

### Mechanical behavior and plastic deformation of the perfect sample

**Figure 4:**
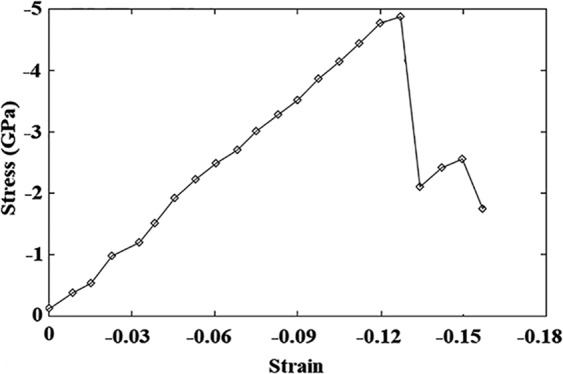
Stress-strain diagram of the perfect nanocomposite model under compression.

At the start of the results part, we’re going to look at the validation test. Accordingly, using the developed LAMMPS code, the perfect copper/silver sample was analyzed at 300 K under uniaxial *compressive* loading conditions. Figure 4 displays the achieved stress-strain diagram that can be utilized to determine the yield stress and Young’s modulus of the sample. It is noted that the former corresponds to the stress level above which, the first plastic deformation occurs within the sample along with a drop in the stress-strain curve. Also, Young’s modulus is defined as the initial slope of the curve in the elastic region. To facilitate compression, the mentioned mechanical properties are summarized in Table 2 along with the available data in the literature. As seen, the results of the pure copper sample are in the range of the corresponding values reported in the literature^41–44^ verifying the developed code. Additionally, comparing the mechanical features of the Cu/Ag nanocomposite model with those of the pure copper sample, it is found that the addition of silver nanoparticle to the base copper would not lead to a remarkable change in the values of the yield strength and Young’s modulus. This is attributed to the similarity of the two phases regarding the crystalline structure and ductility behavior. However, as will be discussed in details, the presence of Ag nanoparticle creates a non-coherent interface, which in turn promotes the plastic deformation of the copper matrix through increasing the structural defects.

**Table 2:** Comparison of the determined mechanical properties of the perfect nanocomposite model with those reported in the literature.

Study	Case	Yield strength (GPa)	Young’s modulus (GPa)
Zhang^41^	Cu single crystal	−4	
Salehinia^42^	Cu single crystal	−5	41
Lin^43^	Cu single crystal	−5.9	
You^44^	Cu single crystal	−3.9	
**Present work**	Cu single crystal	−5.4	41.7
**Present work**	Cu single crystal embedded with Ag NP	−5	38.5

**Figure 5:**
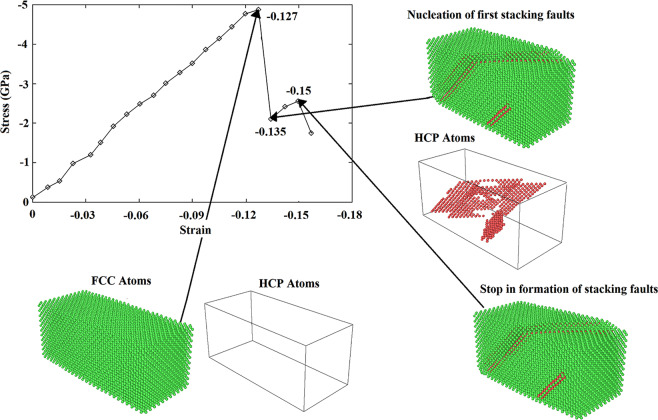
Comparison of the crystal structure variations of the sample before and after the yield point simulated with LAMMPS (version 23Oct2017 https://lammps.sandia.gov/) and visualized using OVITO (version 2.9 27Jul2017 https://www.ovito.org). Atoms are colored based on the CNA method.

It is worth mentioning that as seen in the curve, passing the linear trend observed in the elastic region, there are some fluctuations in the curve and consequently, it follows a zigzag behavior in the plastic region. Since the macroscopic mechanical behavior of materials is ascribed to the structural changes occurred at the atomic-scale, it is necessary to analyze the mentioned zigzag behavior from the crystal structure evolution as depicted in Fig. 5. Employing the CNA approach, it is revealed that before yielding (at the strain of −0.127), the crystal structure is FCC. Passing the yield point and reaching to the strain value of −0.135, first stacking fault regions having HCP crystal structure are formed causing a sudden drop in the curve. Afterward, these regions are developed in the structure without the creation of new stacking faults. This phenomenon would manifest itself by producing the stress increased region during which, the entire sample is kept almost unchanged. Following that, further loading leads to the appearance of new stacking fault regions producing the next drop in the curve. This sequential procedure is repeated in the whole plastic region for the perfect sample embedded with Ag NPs of different sizes and also, samples contain volumetric voids as will be demonstrated in Fig. 11a and Fig. 13, respectively.

**Figure 6:**
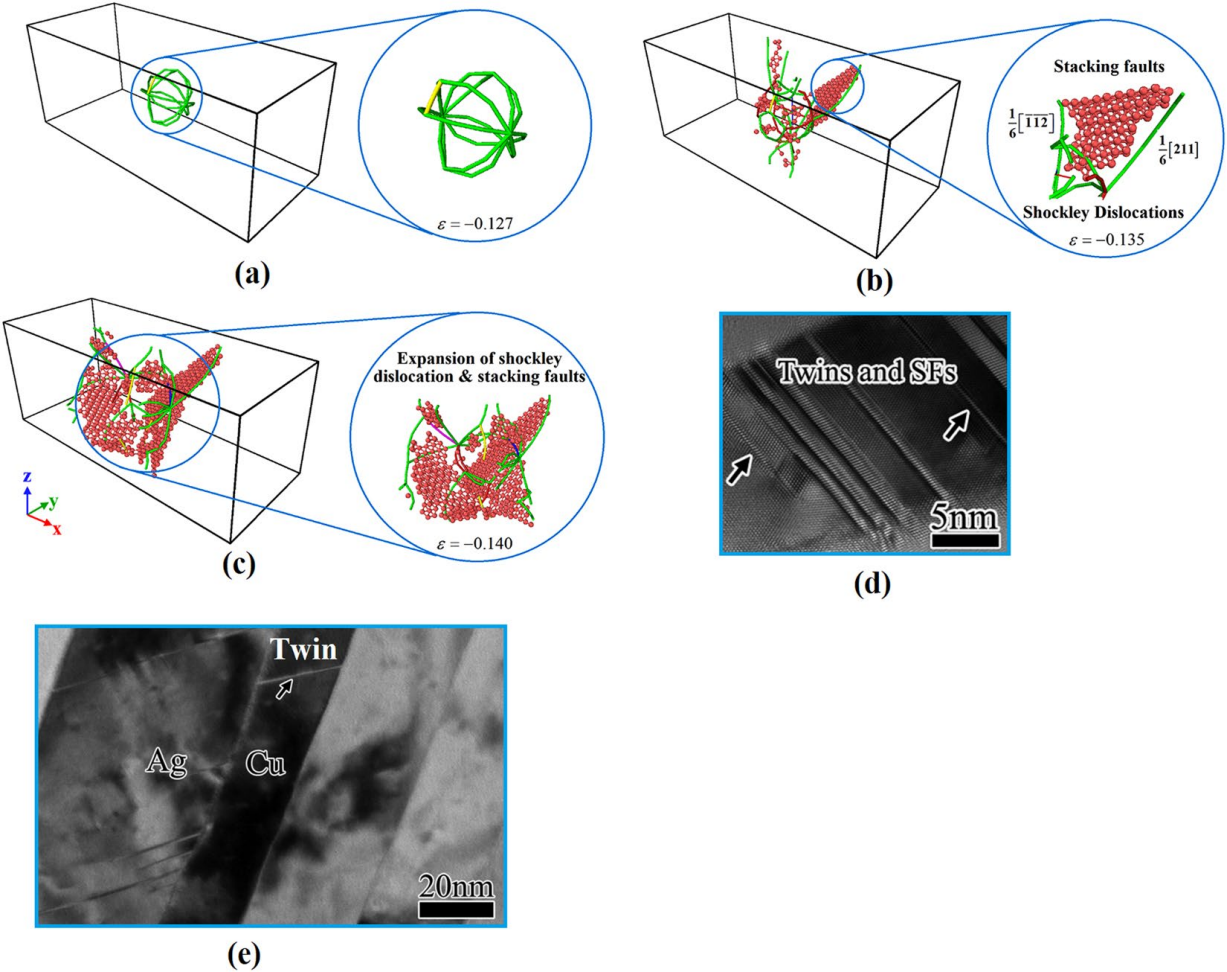
Formation and expansion of Shockley partial dislocations and stacking faults: (**a**) at the onset of yield, (**b**) at the first drop in the stress-strain diagram, and (**c**) after the first decrease in the stress. Configurations have been characterized by the DXA analysis provided by the OVITO program package (version 2.9 27Jul2017 https://www.ovito.org). HRTEM images of: (d) twins and stacking faults in the Ag region and (**e**) a single twin in the copper side of Ag-Cu nanolamellar composites. From ref. ^48^, reproduced with the permission of AIP Publishing.

**Figure 7:**
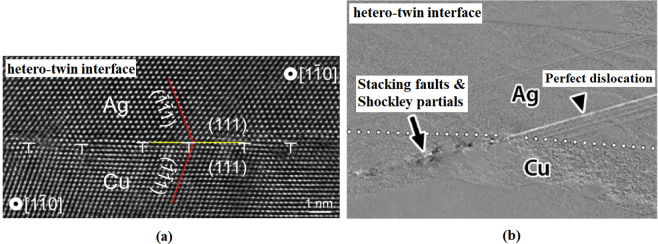
(**a**) TEM image of the hetero-twin interface in Cu-Ag systems. From ref. ^52^, reproduced with the permission of Nature Publishing Group. (**b**): Producing of a perfect dislocation as a result of partial dislocations accommodation in the hetero-twin AgCu interface. From ref. ^53^, reproduced with the permission of Elsevier.

**Figure 8:**
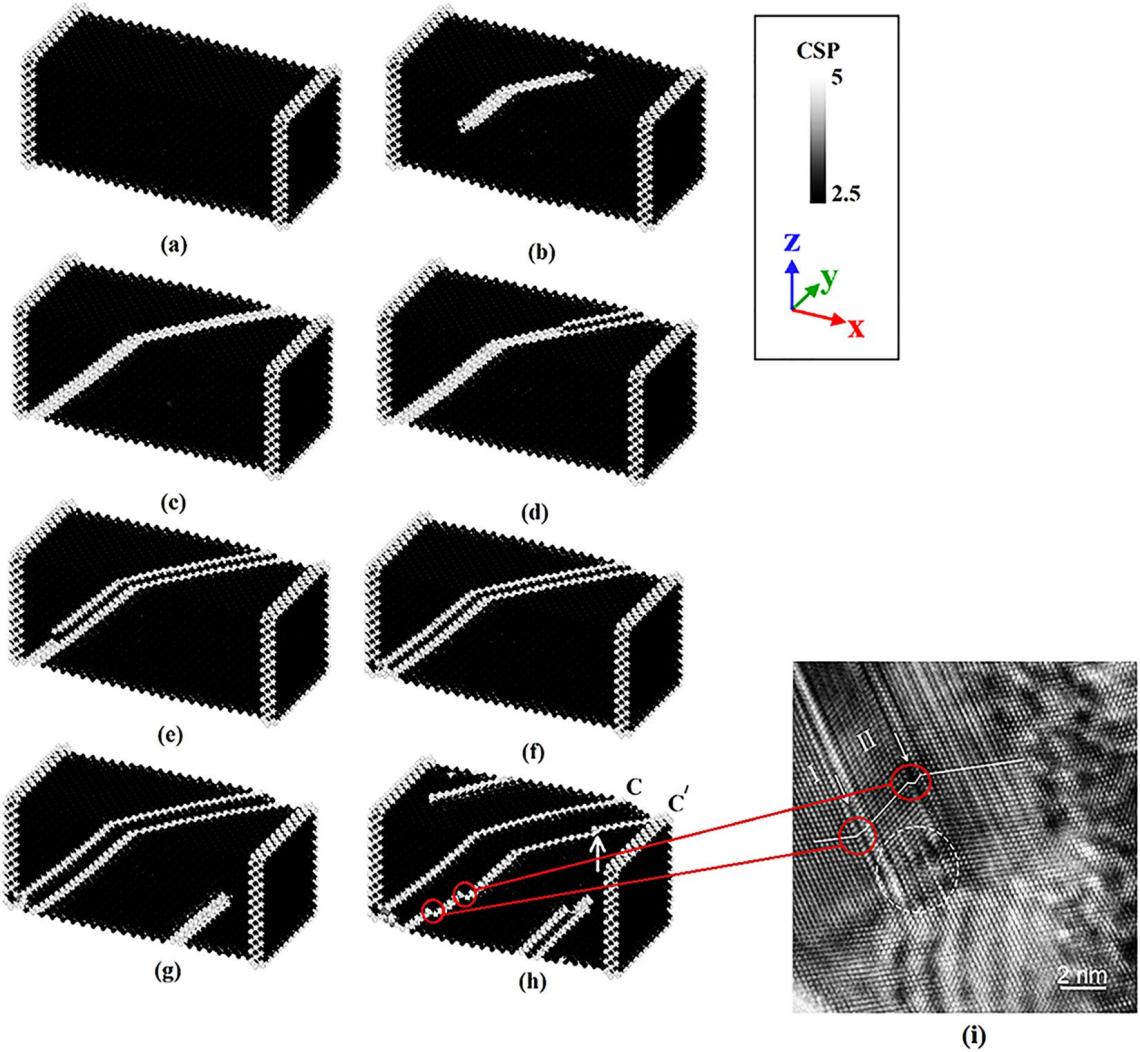
(**a**–**h**) Steps of twin formation from stacking fault regions simulated via LAMMPS (version 23Oct2017 https://lammps.sandia.gov/) and visualized using OVITO (version 2.9 27Jul2017 https://www.ovito.org): (**a**) Stacking faults are created in the interface region, (**b**) Formation of intrinsic stacking faults, (**c**) Expansion of stacking faults, (**d**–**f**) Conversion of intrinsic stacking faults to extrinsic ones, (**g**) Formation of twin, (**h**) Creation of steps in the twin boundary. Atoms are colored based on the CSP approach. (**i**) HRTEM image showing the nucleation of deformation twin during ECAP processing of copper single crystals. From ref. ^56^, reproduced with the permission of AIP Publishing.

**Figure 9:**
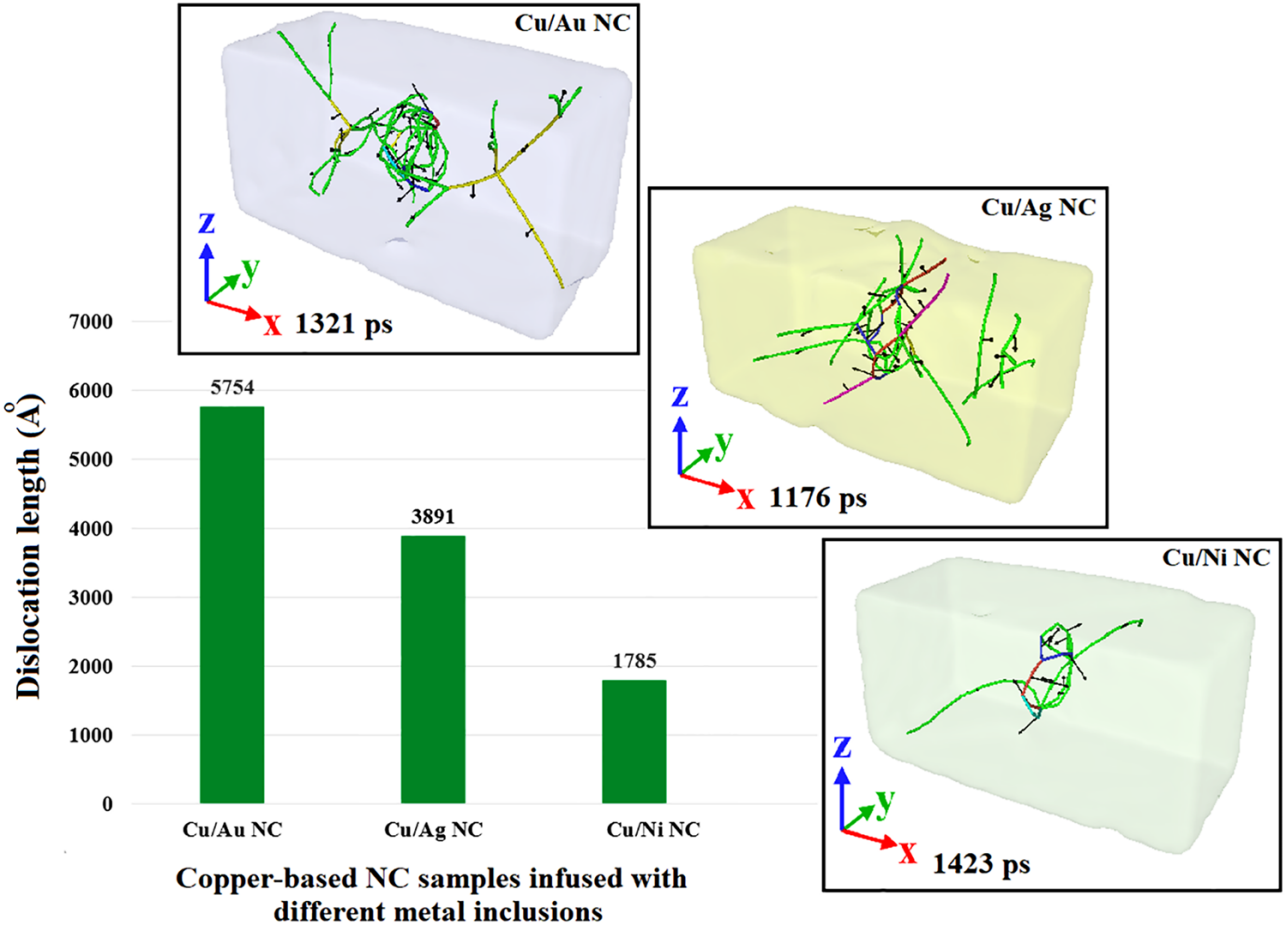
DXA results of molecular dynamics simulation based on LAMMPS (version 23Oct2017 https://lammps.sandia.gov/) representing the effect of interfacial coherency on the emission of Shockley partial dislocations in the interfacial area of different MMNCs. Images have been created employing the OVITO program package (version 2.9 27Jul2017 https://www.ovito.org).

**Figure 10:**
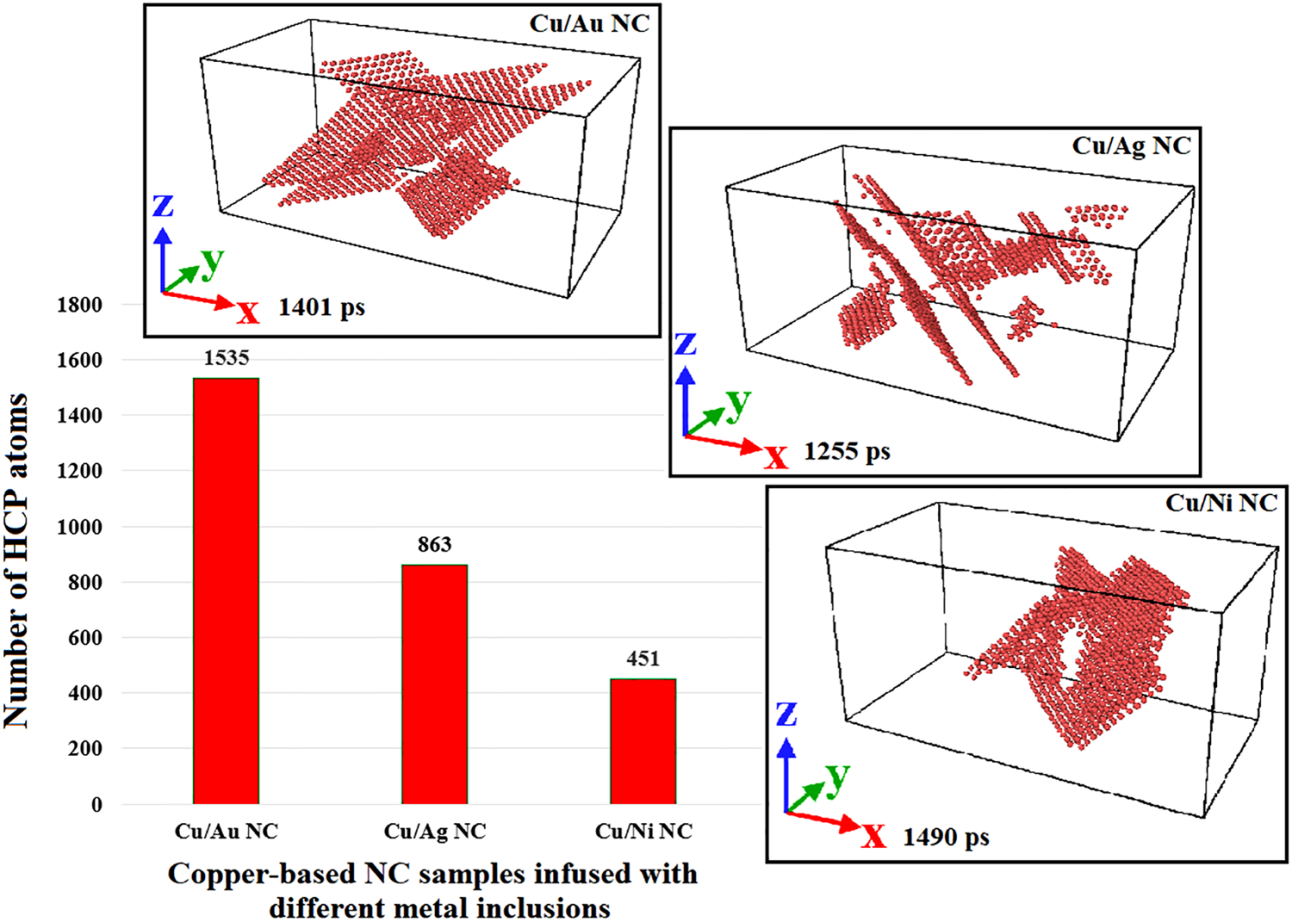
Evolution of the total number of HCP atoms in the copper-based NC samples embedded with different nanoparticles during the whole plastic deformation. Simulations have been carried out in LAMMPS (version 23Oct2017 https://lammps.sandia.gov/) and images have been taken from OVITO (version 2.9 27Jul2017 https://www.ovito.org).

**Figure 11:**
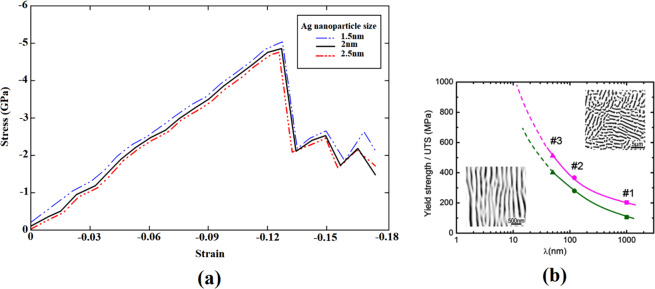
(**a**) Stress-strain curve of the perfect nanocomposite sample infused with Ag NPs of different sizes under compression, (**b**) Variation of the ultimate tensile strength and yield strength of Cu–Ag alloys with the interface thickness (λ). From ref. ^63^, reproduced with the permission of Elsevier.

**Figure 12:**
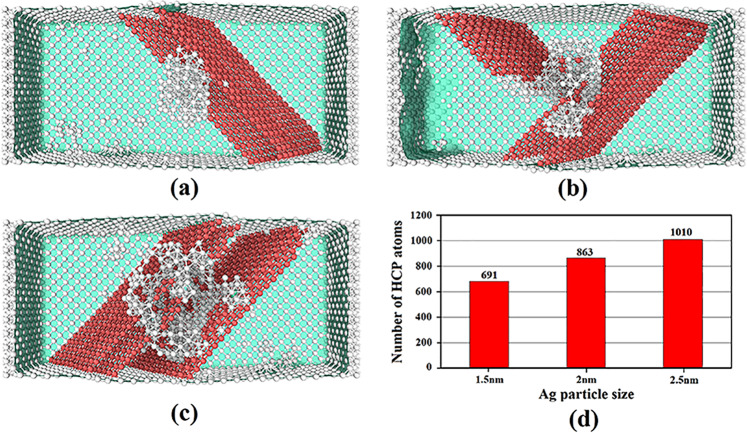
OVITO (version 2.9 27Jul2017 https://www.ovito.org) output representing the cross-section of the copper-based NC samples embedded with Ag NPs of different sizes: (**a**) 1.5 nm, (**b**) 2 nm, (**c**) 2.5 nm. (**d**): Variation of HCP atoms content within these samples.

**Figure 13:**
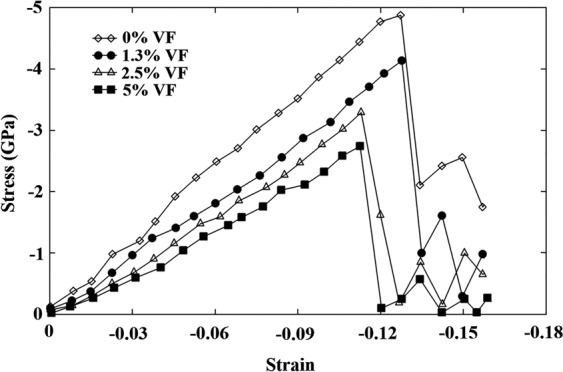
Nanovoids effect on the mechanical response of the NC samples under compression.

The similar zigzag trend was found in the plastic region of copper-silver nanocomposites under tensile loading conditions^34^. This was also observed in the stress-strain diagrams of copper, silver, and nickel samples attributed to the formation of stacking fault regions^45–47^. According to the study carried out by Puksic *et al*.^47^, stacking faults can be directly nucleated on the free surface of nanomaterials. This occurs mainly when the defect-free single crystalline structure is subjected to loading. However, when the crystal structure of the nanomaterial contains geometrical defects, grain boundaries, and second phase with incoherent or semi-coherent interface, stacking faults may form at the other sites in addition to the sample free surface. Furthermore, they can be formed by Shockley partial dislocations as demonstrated by Béjaud *et al*.^45^ via MD simulation of Cu/Ag nanostructured systems. For identification of Shockley dislocations and stacking fault regions in the present study, DXA and CNA techniques were utilized (Fig. 6a–c). As discussed before, passing the yield point, first Shockley partial dislocations nucleate exactly in the interfacial area. Subsequently, their movement to the copper matrix provides a chance for stacking faults to be appeared between these dislocations, which in turn causes a reduction in the curve. This is followed by their expansion due to the movement of partial dislocations producing an increase trend in the curve. This phenomenon was also addressed by An *et al*.^48^ in an experimental study on the Ag-Cu nanolamellar composites. They showed that the twin-based plastic deformation is originated from the emission of Shockley partial dislocations across the interface area. HRTEM images of twins and stacking faults in the Ag region and a single twin in the other part (i.e., copper) have been illustrated in Fig. 6d,e, respectively.

**Figure 14:**
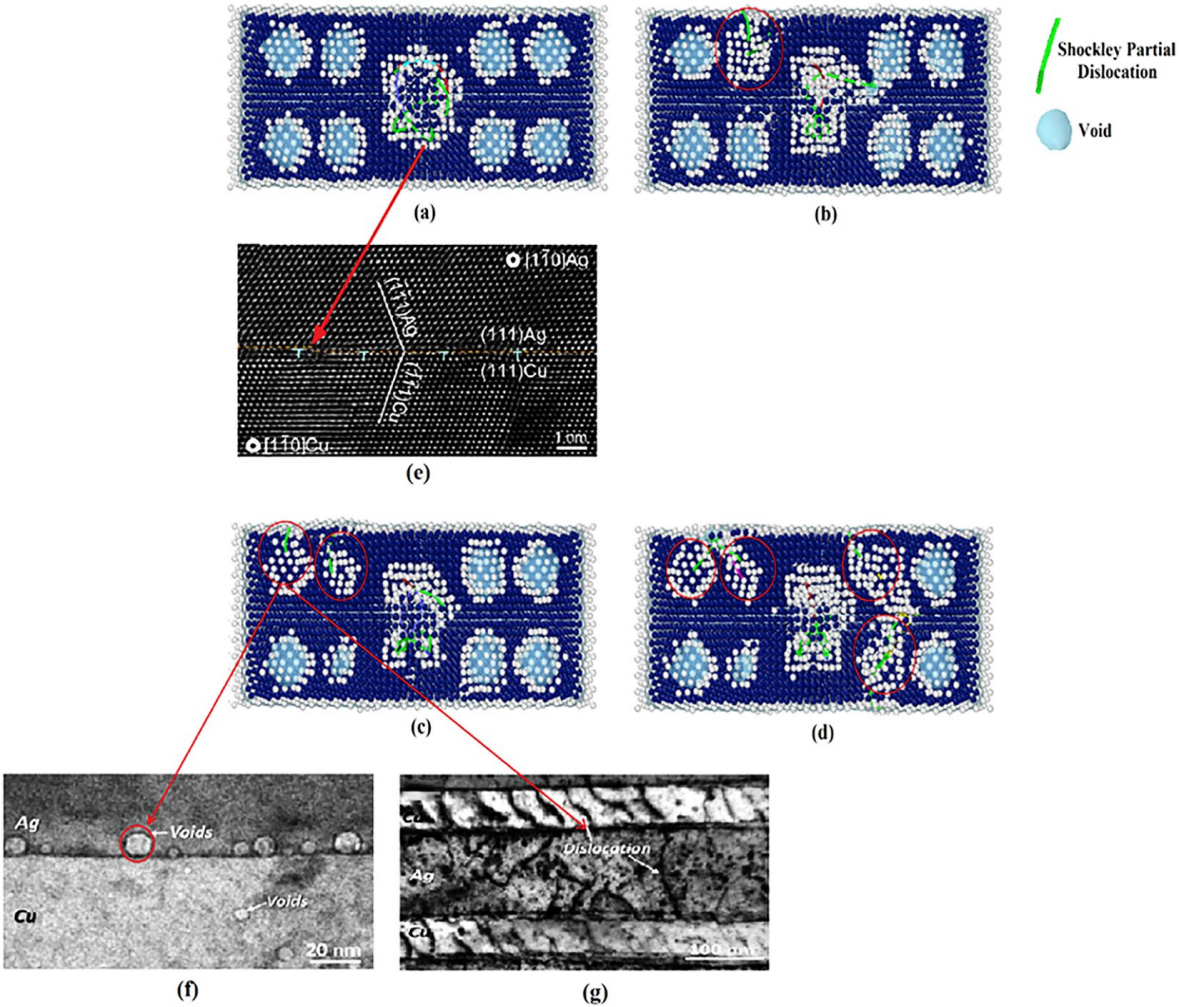
Cross-section of the Cu/Ag NC sample with 2.5% porosity: Nucleation of Shockley partial dislocations from voids and the non-coherent interface at the different strain values of: (**a**) −0.127, (**b**) −0.135, (**c**) −0.139, (**d**) −0.145. Images (**a**–**d**) have been created employing the OVITO program package (version 2.9 27Jul2017 https://www.ovito.org). (**e**): Misfit dislocations at the interfacial area of Cu/Ag sample. From ref. ^66^, reproduced with the permission of Elsevier. (**f**–**g**): Nucleation of partial dislocations from voids at the interface of Cu/Ag NCs. From ref. ^49^, reproduced with the permission of Elsevier.

Therefore, it can be concluded that the copper-silver interface is an important site for the nucleation of Shockley partial dislocations. This is attributed to the 12.5% difference in the atomic radius of copper and silver and thus, an atomic misfit in the interface area as demonstrated in previous MD simulations of this composite system [27, 34, and 45]. This phenomenon leads to misfit dislocations. In addition to the mentioned numerical investigations, it was also experimentally confirmed that the copper-silver interface can facilitate the dislocation nucleation^49^. Porter *et al*.^50^ showed that for the semi-coherent interface, in the absence of external loading conditions, a cohesive strain is created at the interface with a direction opposite to that of the misfit strain to cancel out its effect. This is not the case happened after yielding in which, the matrix and nanoparticles are deformed resulting in the increase of the discussed atomic misfit. Accordingly, the cohesive strain would not be capable of preserving the continuity of the interface. Thus, this area would be a preferred site for dislocation nucleation. This issue has been experimentally confirmed for Cu-Ag systems in the literature^51^. For example, Zheng *et al*.^52^ introduced the Cu/Ag interfacial zone as the hetero-twin (non-coherent) interface, which is not capable of defects movement across the region (see Fig. 7a). Accordingly, owing to the high amount of dislocation interactions in this area, it would be considered as an important site for nucleation of partial dislocation. It was also illustrated that the hetero-twin interface can alter the nature of defects. As disclosed in Fig. 7b, Eftink *et al*.^53^ showed that Shockley partial dislocations and stacking faults could be combined in the Cu/Ag interface to form the perfect dislocation. Using MD simulations, we also found similar non-coherent interface in the afore-mentioned composite systems (See. Figs. 6 and 14).

As discussed before, it has been shown that the plastic deformation of nanostructured copper at the room temperature is usually dominated by twinnings formation that is mainly due to its low SFE. Zhao *et al*.^54^ demonstrated that this phenomenon is initiated by the creation of intrinsic stacking faults between two partial dislocations. Following that, their extrinsic counterparts are introduced within the samples leading to the formation of twinning, based on the so called Pole mechanism^55^. Accordingly, we wanted to check whether this mechanism is working for the perfect nanocomposite sample or not. This was achieved through inspection of the centrosymmetry parameter as depicted in Fig. 8. This factor was limited in the range of 2.5 to 5 for copper and silver system. In this case, the atoms positioned at the stacking fault regions and twins could be distinguished from the rest of atoms^27^. Therefore, the sample shown in Fig. 8a has a similar feature to that of Fig. 6b, in which stacking faults are still within the bulk sample and not on the surface. In Fig. 8b, an intrinsic stacking fault region is formed in ($1\bar{1}\bar{1}$) plane, which is expanded through the whole sample surface as revealed in Fig. 8c. This is followed by conversion of intrinsic stacking faults to extrinsic ones as illustrated in Fig. 8d–f. Twin boundaries can then be observed in Fig. 8g where another stacking fault has been formed.

It is noted that with increasing the stress level, two distinct microstructural changes would be observed within the sample. At first, in this case, the microstructure must sustain more plastic deformation and as a result, the distance between twinning boundaries (c,c’) increases to facilitate the process (See Fig. 8h). Therefore, it is observed that with increasing the compressive stress, migration of twin boundaries leads to the occurrence of the more plastic strain in the sample. The second phenomenon can be perceived by following line c’, which includes several steps as shown in Fig. 8h. Similar deformation twinning has also been experimentally observed by Han *et al*.^56^ during ECAP processing of copper single crystals (See Fig. 8i). As seen in this image, there are some steps created in the twin domains I and II, which is in accordance with our findings observed in red circle in Fig. 8h. Moreover, in line with the mechanism proposed here, their results provide supporting evidence that the twinning formation is highly dependent on the activity of partial dislocations. In this context, Zhu *et al*.^11^ reviewed the experimental studies on the plastic deformation of nanocrystalline FCC metals. It was concluded that the twinning deformation originated from the emission of Shockley partial dislocations is the main mechanism governing their deformation. As discussed in detail in^56^, the mentioned deformation twins would be stopped with microtwins. These important regions depicted by white dashed circle at the lower part of domain II comprise high densities of stacking faults. Similar microtwins were also observed by Liao *et al*.^14^ during the analysis of nanocrystalline copper samples under compressive loading conditions. It is worth mentioning that under tensile loading conditions, no microtwins appear along the twin boundaries^31^.

Therefore, based on the above discussions, it is clear that the twinning formation is main mechanism dominating the plastic deformation of copper-silver nanocomposite systems subjected to *compressive* loadings. Nucleation of Shockley partial dislocations with concurrent formation of stacking fault regions between them has been enumerated as the main reason for the occurrence of the twinning morphology in the copper-based systems^57^. Our findings are in line with the experimental observations of Beyerlein *et al*.^58^, which confirm that the twinning phenomenon in the copper-silver nanocomposite starts from the interface. It should be pointed out that in addition to the interfacial region, the SFE is also an important parameter affecting the plastic deformation mechanism of these NCs. Accordingly, it can be concluded that if we change the role of the constituents in the discussed composite systems, the plastic deformation will be intensified through the enhanced twinning formation. This is attributed to the lower intrinsic SFE of Ag compared to that of Cu as predicted by Hunter *et al*.^59^.

To provide a picture of how the plastic deformation of MMNCs is influenced by the interface, we further proceeded to analyze other samples having different degrees of interfacial coherency. Accordingly, two other copper-based NCs infused with the same amount of Au and Ni NPs (i.e., 2.6% VF) were also examined under the prescribed compressive loading conditions. It is noted that similar to the Cu/Ag sample, the former resembles a case with the semi-coherent interface owing to 12.5% difference between the atomic radius of Cu and Au^60^. It has been proven that this type of interface is formed for each pair of elements having more than 5% lattice mismatch^50^. In contrast, for the Cu/Ni sample, a coherent-like interface is formed due to the only 3.1% atomic mismatch between them^60^. This difference in the interfacial coherency would manifest itself in the emission of partial dislocations nucleated in the interface zone of these composite systems (See Fig. 9). As illustrated in this figure, a semi-coherent interface causes the misfit dislocations to occur more intensely compared to the samples such as Cu/Ni having the coherent-like interface. To facilitate better comparison, snapshots of the samples having the maximum partial dislocation length are also depicted in this figure. These microstructural configurations were characterized by the DXA analysis.

Another important factor leading to the different behavior of these MMNCs is the stacking fault energy. As discussed in the introduction, it has been confirmed that there is a reverse correlation between the SFE and the extent of stacking faults in the metals and metallic systems^61^. To shed more light on this issue, we also approached to explore the role of SFE on the plastic deformation behavior of the introduced samples. Figure 10 depicts the variation of the number of HCP atoms appeared in the stacking fault regions for these systems. Additionally, using the CNA method, snapshots having the most HCP atoms during the whole plastic deformation are illustrated in this figure. In accordance with the data reported in Fig. 9, it is obvious that due to its high SFE (i.e., 99.7 $mJ.{m}^{-2}$^62^), the sample embedded with Ni nanoparticle has the lowest number of HCP atoms. In this case, not only a high density of stacking faults cannot be formed within the Ni inclusion, but also, more importantly, their expansion through the interfacial area and finally, the bulk copper is hard to occur. Contrary to the former case, significant regions having HCP atoms were observed for Cu/Ag and Cu/Au nanocomposites, which is attributed to the low values of SFE reported for silver and gold NPs (i.e., $27.5$ and $18.8\,mJ.{m}^{-2}$, respectively^62^). Accordingly, compared to Ag NPs, the presence of Au inclusion could promote the plastic deformation of the bulk copper through the enhanced twinning formation. However, this mechanism would not be effective in Cu/Ni samples, presumably owing to the lack of sufficient partial dislocations and stacking faults in their interfacial area as the main resources needed for twinning formation.

As observed in the previous discussion, the type of inclusions is very important in determining the mechanism of MMNCs deformation. But, a vital question that should be answered is as follows: What is the size effect of the interfacial region on the deformation twinning? For this purpose, in addition to the introduced sample infused with the 2 nm diameter Ag NP, we further examined the copper systems embedded with Ag nanoparticles of 1.5 and 2.5 nm diameter having the VF of 1.98 and 3.25%, respectively. Figure 11a displays the stress-strain curves of these samples. As seen, increasing the nanoparticle size would lead to reduction of the yield strength. It arises because in the case infused with larger Ag NP, the presence of more interfacial region creates more crystalline defects within the NC sample. The reverse correlation between the size of the interfacial area and the mechanical characteristics of Cu-Ag composite systems has also been experimentally observed by Tian *et al*.^63^. As previously discussed, due to the presence of the hetero-twin interface in these systems, this region would be potentially a preferred site for enhanced deformation twinning. Accordingly, as seen in Fig. 11b, increasing the size of the interface thickness (denoted by ${\rm{\lambda }})$ has led to the reduction of the ultimate tensile strength and yield strength of the samples, which is in line with our MD results. Since twins are originated from the formation of the first stacking fault regions after the yield point, this issue was thoroughly examined as shown in Fig. 12. The microstructure of highly expanded stacking faults demonstrates that larger size of the inclusion causes the deformation twinning to occur more frequently in the nanocomposite systems. To provide a support for this finding, the number of HCP atoms during the whole plastic deformation has been illustrated in Fig. 12d for the introduced samples. It is deduced that there is a direct correlation between the NP size and the extent of stacking faults as the main source of twins formation. It is worth mentioning that the plastic deformation of MMNCs can also be influenced by interaction of the crystalline defects such as twins and dislocations. In this context, Wang and Zhang^64^ demonstrated that this phenomenon could increase the stress required for motions of twins in the nanotwinned metals.

### Role of nanovoids in the proposed mechanism

After examining the perfect Cu/Ag NC sample, the subject was thoroughly inspected in the case of defected samples to find out the role of topological voids on the creation of dislocations, stacking fault regions, twins and in general, mechanical features of the NC sample. For this purpose, three samples containing different voids content were subjected to uniaxial compression loading. Geometrical characteristics of the samples have been listed in Table 1. As disclosed in their stress-strain diagrams (See Fig. 13), increasing the voids content within the sample would cause a substantial reduction in its mechanical charactersitcis. For better comparison, the values representing the Young modulus and yield strength of the porous systems along with those of the defect-free case have been summarized in Table 3. It should be remarked that the detrimental role of these geometrical defects has also been reported under *tensile* loading conditions^32^.

**Table 3:** Weakening effect of the nanovoids content on the yield strength and Young’s modulus of the NC sample under compressive loadings.

Case	Yield strength (GPa)	Young’s modulus (GPa)
Perfect nanocomposite sample	−5	38.5
Sample with 1.3% VF of Volumetric Porosities	−4.7	36.1
Sample with 2.5% VF of Volumetric Porosities	−3.5	31.4
Sample with 5.0% VF of Volumetric Porosities	−2.9	26.3

**Figure 15:**
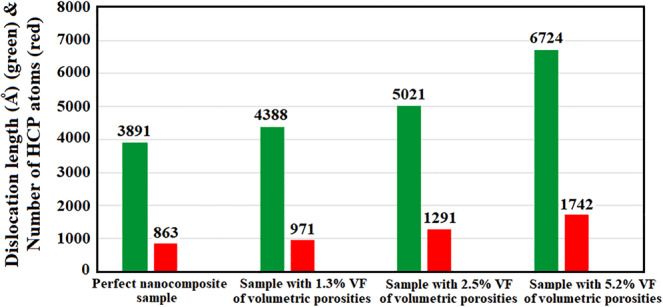
The results of DXA analysis showing the effect of voids content on the partial dislocations length and number of HCP atoms.

Considering the proposed mechanism highlighting the role of twinning formation on the plastic deformation of these nanocomposites, it seems that the presence of voids would affect the creation of Shockley partial dislocations and stacking fault regions as the resources of twinning. This was attributed to the stress concentration around the voids as numerically confirmed by Traiviratana *et al*.^65^. For a more detailed investigation of this issue, the cross-section of the nanocomposite sample containing 2.5% porosity was studied using the dislocation extraction algorithm (See Fig. 14a–d). As seen, with increasing the applied compressive strain, voids act as nucleation sites for Shockley partial dislocations promoting the plastic deformation within the sample. In the last decade, several experimental evidences have uncovered the nucleation of partial dislocations in Cu/Ag nanocomposites. In this content, utilizing HRTEM (See Fig. 14e), Zheng *et al*.^66^ showed that the Cu/Ag interface possesses similar misfit dislocations to those of reported by our MD simulations in Figs. 6b and 14a. Additionally, as illustrated in Fig. 14f, Wang *et al*.^49^ analyzed the defect-interface interactions in the irradiated Cu/Ag NCs including some voids attached to the interface. TEM image shown in Fig. 14g demonstrates that these defects could promote the nucleation of partial dislocations as addressed in Fig. 14c,d of the present work. Therefore, based on the previous experimental observations and the discussed simulation results, it is expected that increasing the voids content would lead to the rise of partial dislocations density and stacking fault regions. To have a *quantitative* tool supporting this idea, the total length of Shockley partial dislocations and the number of stacking fault atoms were obtained for all of the three introduced samples. Figure 15 discloses the results along with those of the perfect sample to facilitate better comparisons. It is revealed that increasing the voids content accelerates the nucleation of partial dislocations. Consequently, since stacking fault regions are formed between two partial dislocations, this phenomenon enhances the number of stacking fault atoms. Accordingly, samples with higher porosities are more prone to form twins. As a result, their plastic deformation is accelerated towards the weakening of the mechanical properties.

## Conclusions

In summary, several MD simulations were conducted to explore the dominant mechanisms affecting the plastic deformation of perfect and defected copper-silver NCs under compressive loadings. It was deduced that at the room temperature and high strain rate conditions, twinning formation is the governing mechanism. It was found that twins are created from the conversion of intrinsic to extrinsic stacking faults. We further proceeded to examine the proposed mechanism in other copper-based composite systems embedded with Au and Ni nanoparticles. The main aim was to assess the effect of interfacial coherency on the nucleation of partial dislocations and the formation of stacking faults as the main resources of twinning-based plastic deformation in MMNCs. Moreover, the results revealed that the presence of voids within the bulk samples provides more active sites for nucleation of Shockley partial dislocations. This is followed by the conversion of stacking faults to twinning, which leads to a significant drop in the mechanical features of the NC samples. It is worth mentioning that to support the main findings of the present research, several experimental-based observations were also provided throughout the article. Since various factors affecting the plastic deformation of MMNCs under compressive loadings have been thoroughly studied in this work, the introduced mechanisms can be used to tailor the mechanical performance of these NCs as medical implants.

## Data Availability

All data are available from the authors upon request.
